# MOTILIPERM Ameliorates Immobilization Stress-Induced Testicular Dysfunction via Inhibition of Oxidative Stress and Modulation of the Nrf2/HO-1 Pathway in SD Rats

**DOI:** 10.3390/ijms21134750

**Published:** 2020-07-03

**Authors:** Keshab Kumar Karna, Kiran Kumar Soni, Jae Hyung You, Na Young Choi, Hye Kyung Kim, Chul Young Kim, Sung Won Lee, Yu Seob Shin, Jong Kwan Park

**Affiliations:** 1 Department of Urology and Research Institute of Clinical Medicine of Jeonbuk National University-Biomedical Research Institute and Clinical Trial Center for Medical Device, Jeonbuk National University Hospital, Jeonju 54907, Korea; karnakeshab@gmail.com (K.K.K.); yjh21111@daum.net (J.H.Y.); cybernyy@naver.com (N.Y.C.); 2 Department of Biological Sciences, Kent State University, Kent, OH 44242, USA; kks2531@gmail.com; 3 College of Pharmacy, Kyungsung University, Busan 48434, Korea; fiona30@ks.ac.kr; 4 College of Pharmacy, Hanyang University, Ansan 426791, Korea; chulykim@hanyang.ac.kr; 5 Department of Urology, Samsung Medical Center, Samsung Biomedical Research Institute, Sungkyunkwan University School of Medicine, Seoul 06351, Korea; drswlee@skku.edu

**Keywords:** stress by immobilization, MOTILIPERM, testis, oxidative stress, apoptosis

## Abstract

It is well established that physiological stress has an adverse effect on the male reproductive system. Experimental studies have demonstrated the promising effects of MOTILIPERM in male infertility. MOTILIPERM extract is composed of three crude medicinal herbs: *Morinda officinalis* How (Rubiaceae) roots, *Allium cepa* L. (Liliaceae) outer scales, and *Cuscuta chinensis* Lamark (convolvulaceae) seeds. The present study aimed to investigate the possible mechanisms responsible for the effects of MOTILIPERM on testicular dysfunction induced by immobilization stress. Fifty male Sprague Dawley rats were divided into five groups (10 rats each): a normal control group (CTR), a control group administered MOTILIPERM 200 mg/kg (M 200), an immobilization-induced stress control group (S), an immobilization-induced stress group administered MOTILIPERM 100 mg/kg (S + M 100), and MOTILIPERM 200 mg/kg (S + M 200). Stressed rats (*n* = 30) were subjected to stress by immobilization for 6 h by placing them in a Perspex restraint cage, while controls (*n* = 20) were maintained without disturbance. Rats were administrated 100 or 200 mg/kg MOTILIPERM once daily for 30 days 1 h prior to immobilization. At the end of the treatment period, we measured body and reproductive organ weight; sperm parameters; histopathological damage; reproductive hormone levels; steroidogenic acute regulatory protein (StAR); biomarkers of oxidative stress; and apoptosis markers. MOTILIPERM treatment improved testicular dysfunction by up-regulating (*p* < 0.05) sperm count, sperm motility, serum testosterone level, StAR protein level, Johnsen score, and spermatogenic cell density in stressed rats. MOTILIPERM decreased oxidative stress by increasing (*p* < 0.05) testicular superoxide dismutase (SOD), glutathione peroxidase (GPx), glutathione peroxidase-4 (GPx 4), catalase, nuclear factor erythroid 2-related factor 2 (Nrf2), and heme oxygenase 1 (HO-1) levels and decreasing (*p* < 0.05) malondialdehyde (MDA) and reactive oxygen species/reactive nitrogen species (ROS/RNS) levels. Furthermore, MOTILIPERM down-regulated (*p* < 0.05) cleaved caspase 3 and BCL2 associated X protein (Bax) levels; increased pro caspase-3 and B-cell lymphoma 2 (Bcl-2) levels; and upregulated testicular germ cell proliferation in stressed rats. The number of terminal deoxynucleotidyl transferase-mediated dUTP nick-end labeling (TUNEL)-positive cells and serum luteinizing hormone (LH) and follicle stimulating hormone (FSH) levels also significantly (*p* < 0.05) decreased after pretreatment with MOTILIPERM in stressed rats. Collectively, our results suggest that, in immobilization-mediated stress-induced testicular dysfunction, MOTILIPERM sustains normal spermatogenesis via antioxidant and anti-apoptotic activities by activating the NRF/HO-1 signaling pathway.

## 1. Introduction

Male reproductive health has markedly deteriorated over the past few decades. In particular, a decline in semen quality around the world has led to an increase in the prevalence of infertility [1]. Infertility can be defined as failure of a couple to conceive after 12 months or more of attempting regular unprotected intercourse [2]. Infertility affects approximately 10–15% of married couples, which represents at least 30 million infertile men among the world’s population [3]. Male infertility has multifactorial causes, such as failure in spermatogenesis, defects in sperm transportation, genetic defects, hormonal dysfunction, aging, and environment and lifestyle factors [4,5,6,7]. Among these factors, physiological and psychological stress appears to be one of the main causes of dysfunction of male reproductive health [8]. A fertile male undergoes a complex process for production of normal, mature spermatozoa by the testis, called spermatogenesis [9]. Decreased sperm count, poor sperm quality, or a combination thereof are evident in 90% of infertile males [5]. Nevertheless, the pathophysiology of idiopathic male infertility still needs to be examined.

In our day-to-day lives, stress has become an omnipresent phenomenon. Stress plays a key role in alterations in various physiological responses and can even lead to various diseases, including sub-fertility or infertility in men [10,11]. Stress has an adverse effect on endocrine function and the male reproductive system, including alterations in androgenic hormone levels, sperm maturation, and testicular function [12]. It has been demonstrated that psychological stress disturbs testicular tight junctions, decreasing seminal quality and germ cell apoptosis [3]. The deleterious effects of chronic stress on reproductive function result in part from the activation and depression of the hypothalamus–pituitary–adrenal (HPA) axis [13]. Stress-induced activation of the HPA axis influences the expression of gonadotropin-releasing hormone (GnRH), luteinizing hormone (LH), and follicle-stimulating hormone (FSH), which leads to a decrease in testosterone levels in males [11]. Another mechanism responsible for testicular dysfunction in stressful conditions is stimulation of lipid peroxidation and reactive oxygen species (ROS) generation [14,15]. Since the testis contains a high level of polyunsaturated fatty acid, it provides an ideal substrate for ROS generation [16]. Immobilization is one of the most potent stress models in the rat and has been used by other researchers to study the effects of physiological stress on male reproductive function [1,3,14]. In the present study, we performed stress by immobilization in Sprague Dawley (SD) rats in order to mimic physiological and psychological stress.

MOTILIPERM is a mixture of three medicinal plants that was formulated to treat male infertility. The components of MOTILIPERM, *Morinda officinalis* How (Rubiaceae) root, *Cuscuta chinensis* Lamark (Convolvulaceae) seed, and *Allium cepa* L. (Liliaceae) outer scales, reportedly possess a wide spectrum of pharmacological activities, such as antioxidant, antinociceptive, and anti-inflammatory activities [8,17,18]. Due to the synergistic effects of polyherbal formulations, MOTILIPERM has a vast advantage over single herbal formulations [19,20]. The marker compounds in MOTILIPERM include monotropein, diacetyl asperulosidic acid, kaempferol 3-*O*-glucoside, quercetin 4′-*O*-glucoside, and quercetin [19,20]. Monotropein and diacetyl asperulosidic acid is a major iridoid glycoside compound from *Morinda officinalis* How (Rubiaceae) root and exhibit actinociceptive, anticlastogenic, and anti-inflammatory activity [21]. Kaempferol 3-*O*-glucoside is major flavonoid from *Cuscuta chinensis* Lamark (Convolvulaceae) seed, which has been widely used to improve male reproductive functions [19]. Quercetin 4′-*O*-glucoside and quercetin is the predominant compound present in *Allium cepa* L. (Liliaceae) outer scales, which has antioxidant and androgenic effects [22]. Recently, we found that MOTILIPERM has a beneficial effect on spermatogenesis after cisplatin, adriamycin, and finasteride treatment in rats [23,24,25]. Administration of MOTILIPERM can increase germ cell proliferation by protecting the testis from oxidative stress, endoplasmic reticulum (ER) stress, and mitochondrial-mediated apoptosis [19,23,24]. Furthermore, treatment with MOTILIPERM in varicocele-induced rat models restores testicular dysfunction by decreasing oxidative stress, ER stress, and germ cell apoptosis and upregulating testosterone synthesis [19,20]. However, the protective effect of MOTILIPERM against stress by immobilization-induced impairment of spermatogenesis and the possible molecular mechanisms thereof have never been documented.

In the present study, we speculated that MOTILIPERM could be used therapeutically to ameliorate physiological and psychological stress-related testicular dysfunction. To test this hypothesis, we used a rat model of stress by immobilization-induced testicular dysfunction and investigated the protective effects of MOTILIPERM. Our data provide evidence that stress by immobilization in the rat increases germ cell apoptosis in the seminiferous tubules. MOTILIPERM treatment decreased LH and FSH levels, restored testosterone levels, and increased epididymal sperm cell count and sperm motility. Collectively, this evidence demonstrates that treatment with MOTILIPERM ameliorates impairments of spermatogenesis mainly through suppression of oxidative stress by activating the nuclear factor erythroid 2-related factor 2/heme oxygenase 1 (Nrf2/HO-1) signaling pathway.

## 2. Results

### 2.1. Body and Organ Weights

The mean body weight and reproductive organ weight are listed in Table 1. The mean body, testis, and epididymis weight in the immobilization-induced stress control group (S) were significantly lower than those of the normal control (CTR) group (*p* < 0.05), but did not differ from the immobilization-induced stress groups administered MOTILIPERM 100 mg/kg (S + M 100) and MOTILIPERM 200 mg/kg (S + M 200). There were no significant differences in seminal vesicle, prostate, or penis weight among the groups.

### 2.2. Analysis of Sperm Parameters and Hormone Levels

**Figure 1:**
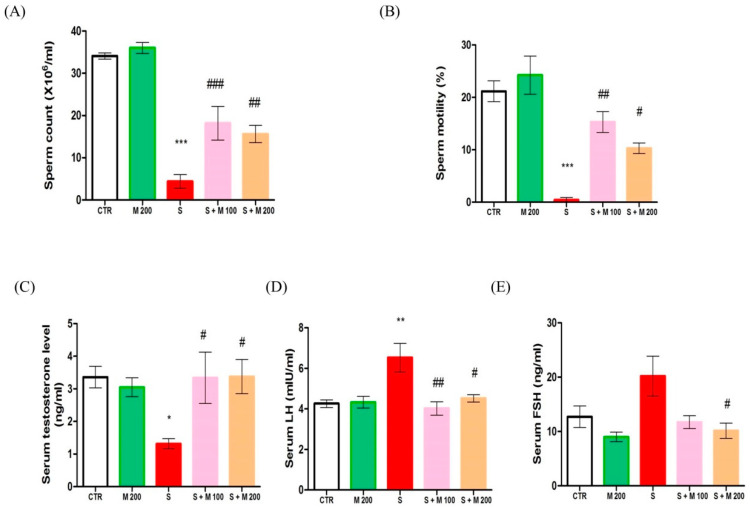
The effect of MOTILIPERM on epididymal sperm count, sperm motility, and serum hormone levels in immobilization stress-treated male SD rats. (**A**) Sperm count in the epididymis. (**B**) Sperm motility in the epididymis. (**C**) Serum testosterone levels. (**D**) Serum luteinizing hormone (LH) levels. (**E**) Serum follicle stimulating hormone (FSH) levels. Data are presented as mean ± SEM. The differences were tested by one-way ANOVA followed by Tukey’s post-hoc test; *n* = 10 for each group. * *p* < 0.05, ** *p* < 0.01, and *** *p* < 0.001 denote significant difference compared to the CTR group; # *p* < 0.05, ## *p* < 0.01, and ### *p* < 0.001 denote significant difference compared to the S group. CTR: control; M 200: MOTILIPERM 200 mg/kg p.o.; S: stress by immobilization group; S + M 100: stress by immobilization + MOTILIPERM 100 mg/kg p.o.; S + M 200: stress by immobilization + MOTILIPERM 200 mg/kg p.o.; p.o.: per oral; ANOVA: analysis of variance; SEM: standard error of the mean.

Mean sperm counts, percentage of sperm motility in the epididymis, and reproductive hormone levels (T, LH, and FSH) in serum are presented in Figure 1. Sperm count, sperm motility, and serum T level in the S group were significantly decreased compared to the CTR group (*p* < 0.05). Conversely, these parameters were markedly increased after pretreatment with 100 mg and 200 mg MOTILIPERM in immobilization-stressed rats (*p* < 0.05). The serum LH level in the S group was significantly higher than that in the CTR group (*p* < 0.05). The serum FSH level was higher in stressed rats compared to CTR rats, but the difference was not statically significant. Pretreatment with MOTILIPERM significantly reduced (*p* < 0.05) serum LH and FSH levels in rats subjected to stress by immobilization.

### 2.3. Histopathological Analysis and Germ Cell Apoptosis

**Figure 2:**
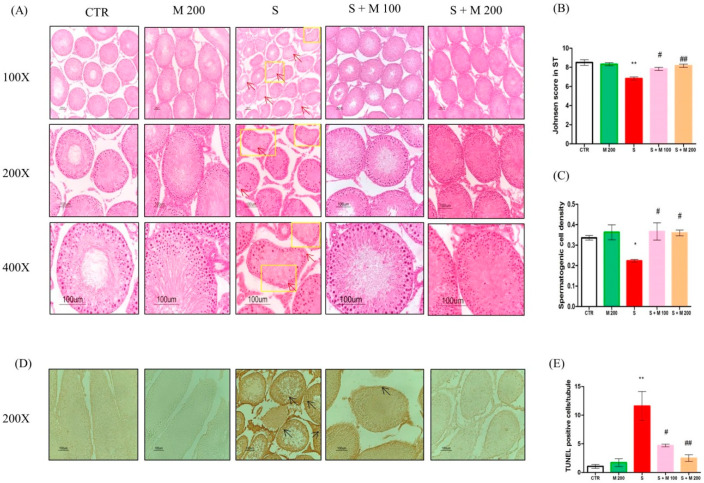
Effect of MOTILIPERM on testicular histopathology and germ cell apoptosis based on hematoxylin and eosin (H&E) staining and terminal deoxynucleotidyl transferase-mediated dUTP nick-end labeling (TUNEL) staining of immobilization stress-treated male SD rats. (**A**) Rat testis cross sections stained with H&E showing degenerating round spermatids, spermatocytes, an absence of spermatozoa, irregular seminiferous tubules, and seminiferous tubules with vacuoles in the S group (arrowhead; a). (**B**) Johnsen’s score in seminiferous tubules. (**C**) Spermatogenic cell density in seminiferous tubules. (**D**) Cross section of TUNEL-stained testis showing TUNEL-positive cells (arrows; d). (**E**) Quantification of TUNEL-positive cells expressed as total positive cells/seminiferous tubule. Data are presented as mean ± SEM. The differences were tested by one-way ANOVA followed by Tukey’s post-hoc test; *n* = 10 for each group. * *p* < 0.05 and ** *p* < 0.01 denote significant difference compared to the CTR group; # *p* < 0.05 and ## *p* < 0.01 denote significant difference compared to the S group. CTR: control; M 200: MOTILIPERM 200 mg/kg p.o.; S: stress by immobilization group; S + M 100: stress by immobilization + MOTILIPERM 100 mg/kg p.o.; S + M 200: stress by immobilization + MOTILIPERM 200 mg/kg p.o.; p.o.: per oral; ANOVA: analysis of variance; SEM: standard error of the mean.

Testicular histomorphological changes and apoptosis of germ cells are presented in Figure 2. The CTR and M 200 groups showed normal spermatogenesis. Proper morphological appearance with all stages of spermatogenesis from spermatogonia to spermatozoa, Sertoli cells, and Leydig cells was observed in almost all seminiferous tubules of the CTR and M 200 groups. In contrast, histological image analysis revealed degenerating round spermatids and spermatocytes, an absence of spermatozoa, and irregular seminiferous tubules with vacuolization in the S group. However, pretreatment with MOTILIPERM in immobilization-stressed rats restored the morphological appearance in the S + M 100 and S + M 200 groups (Figure 2A). As shown in Figure 2B,C, Johnsen’s score and spermatogenic cell density in the S group were significantly reduced compared to the CTR group (*p* < 0.05). Pretreatment with MOTILIPERM significantly ameliorated these changes (*p* < 0.05). The number of terminal deoxynucleotidyl transferase-mediated dUTP nick-end labeling (TUNEL)-positive cells in the S group was markedly increased (*p* < 0.05) compared to those in the CTR group (Figure 2D,E). However, the number of TUNEL-positive cells in the S + M 100 and S + M 200 groups significantly decreased as compared with the S group (*p* < 0.05).

### 2.4. Estimation of Malondialdehyde (MDA), ROS/reactive nitrogen species (RNS), and Enzymatic Antioxidant Levels

**Figure 3:**
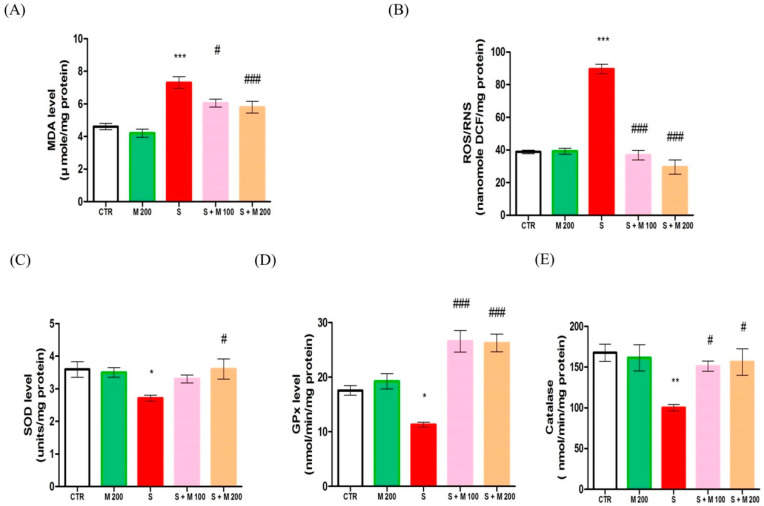
MOTILIPERM treatment attenuates oxidative stress in testis tissue of immobilization stress-treated male Sprague Dawley rats. (**A**) Malonaldehyde levels, (**B**) reactive oxygen species (ROS)/reactive nitrogen species (RNS) levels, (**C**) superoxide dismutase (SOD) levels, (**D**) glutathione peroxidase (GPx) levels, and (**E**) catalase levels. Data are presented as mean ± SEM (*n* = 10 per group). Differences were tested by one-way ANOVA followed by Tukey’s post-hoc test; *n* = 10 per group. * *p* < 0.05 and ** *p* < 0.01 denote significant difference compared to the CTR group; # *p* < 0.05 and ## *p* < 0.01 denote significant difference compared to the S group. CTR: control; M 200: MOTILIPERM 200 mg/kg p.o.; S: stress by immobilization group; S + M 100: stress by immobilization + MOTILIPERM 100 mg/kg p.o.; S + M 200: stress by immobilization + MOTILIPERM 200 mg/kg p.o.; p.o.: per oral; ANOVA: analysis of variance; SEM: standard error of the mean.

MDA, ROS/RNS, and enzymatic antioxidant activity in testis tissue are presented in Figure 3. Stress by immobilization in rats induced significant increases (*p* < 0.001) in the levels of MDA and ROS/RNS compared to CTR treatment. The use of 100 mg or 200 mg MOTILIPERM restored the levels of MDA and ROS/RNS in stressed rats. Furthermore, levels of the antioxidant enzymes superoxide dismutase (SOD), glutathione peroxidase (GPx), and catalase were significantly decreased (*p* < 0.05) in the S group compared to the CTR group. Treatment with MOTILIPERM in stressed rats produced a significant increase (*p* < 0.05) in testicular SOD, GPx, and catalase levels compared with those seen in the S group.

### 2.5. Western Blot and Immunohistochemistry Studies of Protein Expression in Testis Tissue

**Figure 4:**
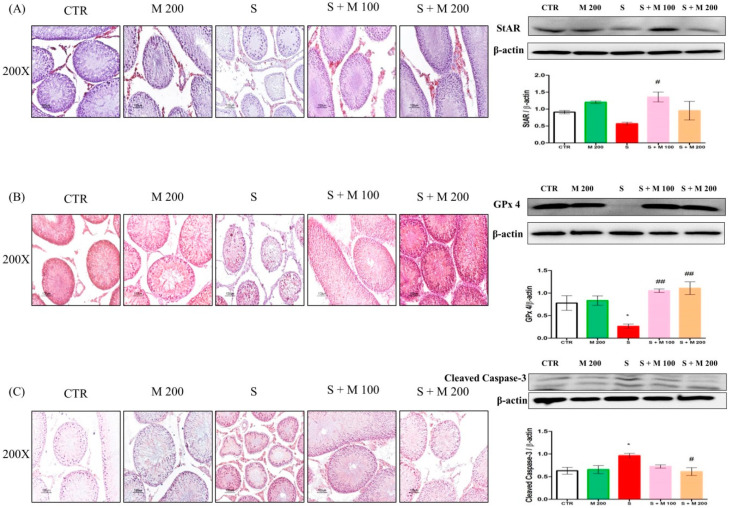

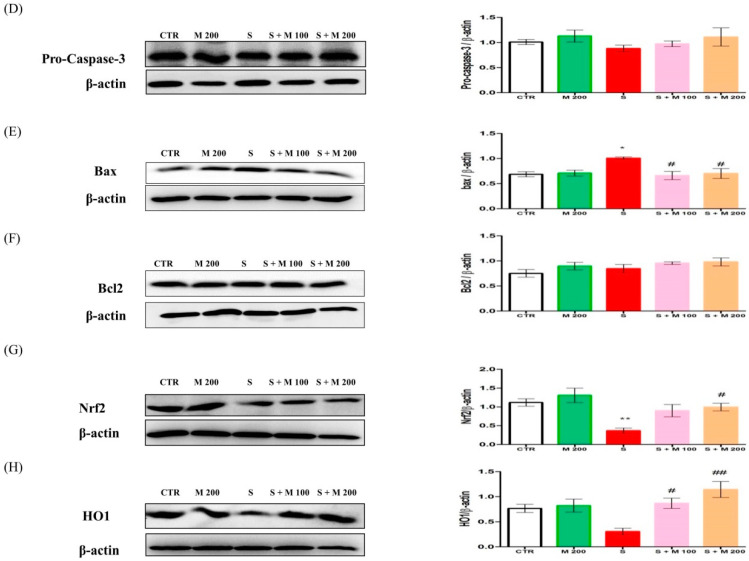
Effect of MOTILIPERM on testicular protein levels in immobilization stress-treated male Sprague Dawley rats determined by Western blot analysis and immunohistochemical staining. (**A**) Steroidogenic acute regulatory protein (StAR), (**B**) glutathione peroxidase-4 (GPx 4), (**C**) cleaved caspase-3, (**D**) Pro-caspase-3, (**E**) BCL2 associated X protein (Bax), (**F**) B-cell lymphoma 2 (Bcl2), (**G**) Nuclear factor erythroid 2-related factor 2 (Nrf2), and (**H**) Heme oxygenase 1 (HO-1). Data are presented as mean ± SEM. The differences were tested by one-way ANOVA followed by Tukey’s post-hoc test; *n* = 10 per group. * *p* < 0.05 and ** *p* < 0.01 denote significant difference compared to the CTR group; # *p* < 0.05 and ## *p* < 0.01 denote significant difference compared to the S group. CTR: control; M 200: MOTILIPERM 200 mg/kg p.o.; S: stress by immobilization group; S + M 100: stress by immobilization + MOTILIPERM 100 mg/kg p.o.; S + M 200: stress by immobilization + MOTILIPERM 200 mg/kg p.o.; p.o.: per oral; ANOVA: analysis of variance; SEM: standard error of the mean.

To analyze the protective mechanism of MOTILIPERM against immobilization stress-induced testicular dysfunction, the protein expression levels of pro-caspase-3, cleaved caspase-3, B-cell lymphoma 2 (Bcl-2), BCL2 associated X protein (BAX), glutathione peroxidase-4 (GPx-4), Nrf2, HO-1, and steroidogenic acute regulatory protein (StAR) were evaluated in testis tissue (Figure 4). Immobilization stress in rats resulted in significant increases (*p* < 0.05) in cleaved caspase-3 and BAX and decreases in GPx 4, Nrf2, HO-1, and StAR levels. There were no significant differences in the protein expression levels of pro-caspase-3 or Bcl2 among all groups. Pretreatment with MOTILIPERM significantly decreased the levels of cleaved caspase-3 and BAX and increased the levels of Gpx4, Nrf2, HO-1, and StAR in testicular tissue. Furthermore, cell-specific expression of StAR, Gpx4, and cleaved caspase 3 in testis tissue were analyzed by immunohistochemical staining (Figure 4). A dark red positive signal associated with StAR was detected in the Leydig cells of the CTR group and the M 200 group. However, StAR protein expression in the S group was diminished. In contrast, pretreatment with MOTILIPERM upregulated the expression of StAR to dark red in the Leydig cells of stressed rats. Dark red Gpx4 immunoreactivity was detected in the seminiferous tubules in the CTR and M 200 groups. In the testis of the S group, reduced immunoreactivity was observed. In contrast, pretreatment with MOTILIPERM upregulated the expression of Gpx4 in the S + M 100 and S + M 200 groups. Similarly, deep brown staining corresponding to cleaved caspase 3 was observed in the S group and was not present in the other groups.

## 3. Discussion

During recent decades, an increase in human infertility that may stem from physiological and psychological stress has gained widespread attention. Stress-induced testicular dysfunction and corresponding pathological features have been well documented in rats [26]. MOTILIPERM has been used as an antioxidant to reduce lipid peroxidation, scavenge free radicles, and prevent germ cell apoptosis in varicoceles and drug-induced testicular toxicity [19,20,23,24,25]. The present study reported for the first time the effect of MOTILIPERM on reproductive parameters in immobilization stress-induced male infertility in adult SD rats.

Our results indicate that the S group showed significant decreases in body, testis, and epididymis weight compared with the CTR group, which is consistent with previous findings [1,27]. Decreases in testicular and epididymis weight may be due to structural changes and a decrease in androgen biosynthesis. The S group showed a decline in sperm count and motility as well as a sloughing off of germ cells and seminiferous tubular atrophy. The reduction in sperm count and motility may stem from increased formation of ROS [28]. Johnsen’s score and spermatogenic cell density were downregulated in the S group compared to the CTR group. The decrease in germ cell number was due to downregulation of testosterone levels. These results are consistent with our previous findings [29]. This study demonstrates that MOTILIPERM restores seminiferous tubular structure, protects germ cell proliferation, and improves sperm count and sperm motility. Likewise, in previous findings, immobilization stress was associated with altered androgen biosynthesis accompanied by higher levels of serum LH and FSH that coincided with a reduction in testosterone [30]. This may be due to changes in the pituitary–Leydig cell axis. Treatment with MOTILIPERM upregulated testosterone levels and ameliorated the increases in FSH and LH to basal levels, which suggests that MOTILIPERM has pro-androgenic activity. Moreover, the StAR protein expression level was significantly lower in the S group; similar findings were observed in previous studies [11]. StAR protein regulates transportation of free cholesterol from the cytoplasm into the mitochondrial inner membrane and converts cholesterol to pregnenolone in the mitochondria via cytochrome P450 side-chain cleavage enzymes [31]. Pregnenolone will be further transported to the smooth endoplasmic reticulum for testosterone and other steroid hormone synthesis [32,33]. Upregulation of StAR protein expression in the testis after pretreatment with MOTILIPERM in stressed rats further confirms the pro-androgenic property of this herbal composition.

In the present study, immobilization induced oxidative stress by increasing lipid peroxidation and free radical generation as well as decreasing antioxidant levels in testicular tissue. Several studies have reported similar findings in stress-induced testicular tissue [12]. Oxidative stress is known as a prime contributor to immobilization stress-induced testicular germ cell apoptosis [15,34]. The decrease in antioxidant enzyme levels is due to their inactivation by overproduction of ROS [35,36]. Antioxidant enzymes scavenge free radicals and prevent oxidative damage by enhancing cellular defenses [37]. ROS can cause extensive damage to DNA, proteins, and lipids in germ cells [38,39]. Our results show that MOTILIPERM decreases the levels of ROS/RNS and MDA and increases levels of the antioxidant enzymes SOD, GPx, catalase, and GPx 4. The protective effect of MOTILIPERM may be attributable to the amelioration in testicular oxidative status, as reported previously [19,23].

Nrf2 (nuclear factor erythroid 2–related factor 2) is a known redox-responsive transcription factor that plays a vital role in preventing oxidative stress by maintaining intracellular redox states [40]. In several cell types, the cellular response to oxidative stress for induction of phase II antioxidant enzymes through transcriptional activation is mediated by antioxidant-response elements (AREs) and Nrf2 [41]. Under oxidative stress conditions, NRF2 dissociates from the Nrf2-Keep1 (Kelch-like ECH associating protein 1) complex and accumulates in the nucleus and binds with the ARE promoter, thereby regulating cytoprotective genes, including HO-1, catalase, SOD, glutathione peroxidase (GSH-PX), and glutathione sulfhydryl transferase (GST) [42]. The present study demonstrates an Nrf2/HO-1 pathway-mediated antioxidant response following MOTILIPERM treatment in stressed rats. Nrf2 plays a direct role in induction of HO-1, a rate-limiting enzyme that catalyzes the degradation of heme into carbon monoxide, ferritin, and biliverdin [43]. Bilirubin and carbon monoxide exhibit anti-oxidant activities. HO-1 plays an important role in maintaining cellular homeostasis and increasing expression of HO-1 is a protective mechanism against oxidative stress [44]. Recent studies have provided evidence for the therapeutic potential of targeting the Nrf2/HO-1 pathway in immobilization stress-induced testicular dysfunction [1,45]. These findings are consistent with our results. In our study, the expression levels of Nrf2, catalase, SOD, and HO-1 were downregulated after stress by immobilization, indicating that immobilization induced testicular dysfunction through oxidative stress. MOTILIPERM treatment upregulated these parameters in stressed rats, further confirming the antioxidant effects of MOTILIPERM.

**Figure 5:**
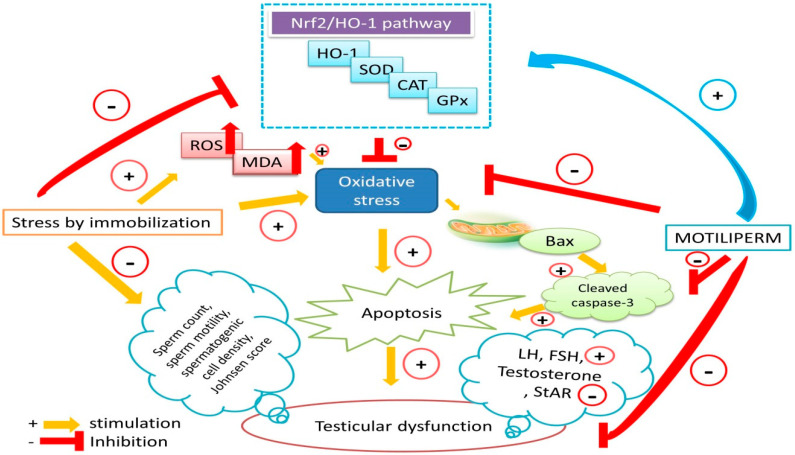
Schematic diagram of the therapeutic effect of MOTILIPERM against immobilization stress-induced testicular dysfunction. Bax: BCL2 associated X protein; Nrf2: nuclear factor erythroid 2-related factor 2; HO-1: heme oxygenase 1; GPx: glutathione peroxidase; SOD: superoxide dismutase; CAT: catalase; ROS: reactive oxygen species; MDA: malonaldehyde; LH: luteinizing hormone; FSH: follicle stimulating hormone.

The present investigation also showed that testicular apoptosis is significantly increased in stressed rats, which is consistent with previous findings [1,3,11,15,29,45]. Bax is an important pro-apoptotic gene; Bcl-2 is an anti-apoptotic gene; and cleaved caspase-3 is an executioner caspase that is essential for triggering apoptosis [24]. Upregulation of cleaved caspase-3 and Bax and downregulation of Bcl2 in the stressed rat suggest germ cell apoptosis via the mitochondrial apoptosis pathway [32]. Furthermore, an increased number of TUNEL-positive cells was seen in the seminiferous tubules, demonstrating that stress by immobilization significantly increases apoptosis in testis tissue. Oxidative stress is well known as a key factor that induces germ cell apoptosis in the testis [46]. Nrf2 over-expression interrupts apoptosis-related events through increasing expression of Bcl-2 and suppression of BAX and cleaved caspase-3 [47]. Pretreatment with MOTILIPERM reversed testicular germ cell apoptosis in stressed rats by upregulating expression of Bcl2, Nrf2, and HO-1 and downregulating TUNEL-positive cells, cleaved caspase-3 and BAX levels (Figure 5).

## 4. Materials and Methods

### 4.1. Plant Material and Extract Preparation

MOTILIPERM extract is composed of three crude medicinal plants: *Morinda officinalis* How (Rubiaceae) roots, *Allium cepa* L. (Liliaceae) outer scales and *Cuscuta chinensis* Lamark (convolvulaceae) seeds. MOTILIPERM was provided by the Herbarium Unit of Hanyang University (herbarium unit voucher number: Cinthera-1, HYUP-MO-001; Cinthera-2, HYUP-CC-001; and Cinthera-3, HYUP-AC-001). Radical scavenging activities of each component for MOTILIPERM were described in Supplementary Materials (Table S1). Crude MOTILIPERM extract was prepared as described previously [19].

### 4.2. Identification of Major Compounds

**Figure 6:**
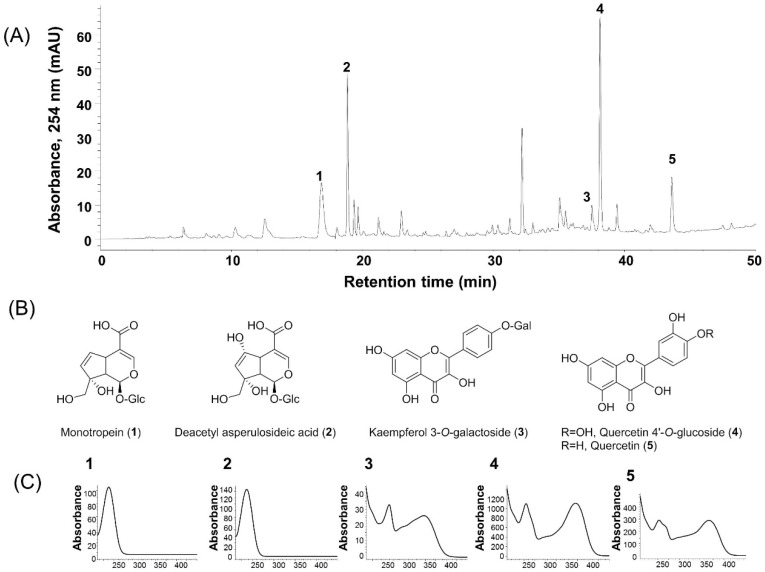
(**A**) High performance liquid chromatography (HPLC) profile of MOTILIPERM. (**B**) Chemical structure of major compounds. Monotropein (**1**), deacetylasperulosidic acid (**2**), kaempferol 3-O-galactoside (**3**), quercetin 4′-O-glucoside (**4**), and quercetin (**5**). (**C**) UV spectra of major compounds.

The major compounds in MOTILIPERM were identified by high-performance liquid chromatography (HPLC) as described previously (Figure 6) [19]. Briefly, the HPLC system used for the analysis was an Agilent 1200 HPLC system (Agilent Technologies Inc, Santa Clara, CA, USA) with an INNO column C18 (4.6 × 250 mm, 5 µm) (YoungJin Biochrom, Seoul, South Korea). Acetonitrile containing 0.1% trifluoroacetic acid (solvent A) and water containing 0.1% trifluoroacetic acid (solvent B) were used as mobile phases with a linear gradient as follows: 0–0% A for 0–10 min, 0–30% A for 10–40 min, 30–50% A for 40–50 min, 50–100% A for 50–60 min, and 100% A for 60–70 min. The injection volume was 10 µL and the chromatographic profile was recorded at wavelengths of 240 and 254 nm with a spectrum ranging from 210 to 450 nm.

### 4.3. Animal Care and Experimental Design

**Figure 7:**
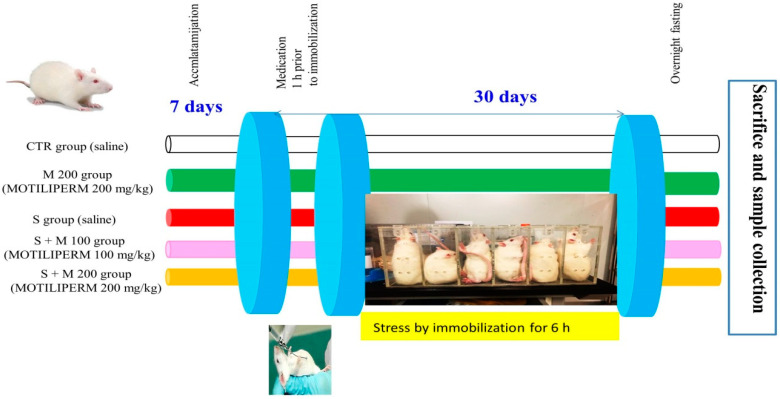
Schematic diagram of the study design. Physiological stress was induced in male Sprague Dawley rats by immobilization in a Perspex restraint cage. CTR: control; M 200: MOTILIPERM 200 mg/kg p.o.; S: stress by immobilization group; S + M 100: stress by immobilization + MOTILIPERM 100 mg/kg p.o.; S + M 200: stress by immobilization + MOTILIPERM 200 mg/kg p.o.

A total of 50 adult male Sprague Dawley rats (240–250 g, 8 weeks old) were purchased from Koatech (Gyeonggi-do, South Korea). The animals were housed in groups of four rats per plastic cage (47 × 18 × 40 cm); maintained under a reversed 12 h light–dark cycle at constant temperature (20 ± 2 °C) and relative humidity (50 ± 10%); fed a diet of standard rat chow; and given free access to water ad libitum. Handling of the rats was done in accordance with the Institutional Animal Care and Use Committee of Jeonbuk National University Hospital Laboratory Animal Center (cuh-IACUC-2017-13). After one week of acclimatization, the total of fifty rats were randomly divided into five groups (ten rats/group): (1) normal control (CTR) group; (2) MOTILIPERM 200 mg/kg p.o. group (M 200); (3) immobilization stress (S) group; (4) S + M 100 mg/kg p.o. group (S + M 100) and (5) S + M 200 mg/kg p.o. group (S + M 200). The animals in the stress groups were subjected to stress by immobilization (IMB) by keeping them in a Perspex restraint cage (6 cm wide × 7cm height × 18 cm long) for 6 h per day during the light period starting at 9 a.m. each day for 30 days (Figure 7) [27]. The CTR and M 200 groups were kept in standard cages. During the stress period, water and food were withdrawn from all groups. MOTILIPERM was dissolved in normal saline and administrated orally at 100 mg/kg or 200 mg/kg in a single dose each day to the rats in the M 200, S + M 100, and S + M 200 groups for 30 days. The dosages of MOTILIPERM (100 and 200 mg/kg) were selected based on a previous study [23]. CTR and S group rats were treated with the same volume of normal saline (vehicle). Medication was administered to the rats 1 h prior to immobilization. After 30 days of treatment, anesthesia was performed using a mixture of ketamine (100 mg/mL) and 2% rumpin (20 mg/mL) in sterile conditions [24]. Rat blood was collected from the venae cavae. Serum was separated and stored at −80 °C until biochemical analysis. Reproductive organs were weighted and rats were killed by exsanguination. Testis tissue was collected immediately, placed in Bouin’s solution, and frozen in liquid nitrogen for further analysis.

### 4.4. Assessment of Sperm Count and Sperm Motility

The total sperm count and percentage of sperm motility were determined as described previously [20]. In brief, the distal cauda epididymis was excised and transferred to a 1.5 mL microcentrifuge tube containing normal saline, nicked at two sites with scissors. Spermatozoa were allowed to suspend in pre-warmed normal saline for 5 min at 37 °C. The total sperm count was calculated by counting the number of sperm in 10 squares of the grid after adding two drops of each specimen onto a counting chamber (SEFI-Medical Instruments Ltd., Hicksville, NY, USA) under an Axio Imager 2 light microscope (Carl Zeiss MicroImaging LLC, Goettingen, Germany) at 20× magnification. Sperm count is expressed as 10^6^ sperms/mL. Sperm motility was evaluated under a light microscope at 20× magnification within 10 squares of the grid on a pre-warmed counting chamber. The percentage of motile spermatozoa was analyzed by the formula ((mean number of motile spermatozoa/total number of spermatozoa) × 100) and the results are given as a percentage.

### 4.5. Measurement Of Testosterone, Luteinizing Hormone, and Follicle-Stimulating Hormone

The concentrations of testosterone (ab108666, Abcam, Cambridge, MA USA), luteinizing hormone (LH) (E-EL-R0026, Elabscience, Houston, TX, USA), and follicle-stimulating hormone (FSH) (E-EL-R0391, Elabscience, Houston, TX, USA) in serum were measured using a commercial enzyme-linked immunosorbent assay (ELISA) kits as per the manufacturer’s protocols.

### 4.6. Testicular Histological Studies and Terminal Deoxynucleotidyl Transferase-Mediated (dUTP) Nick-End Labeling (TUNEL) Staining

Testis tissue fixed in Bouin’s solution was dehydrated, paraffin wax embedded, sectioned to 5 μm, and stained with hematoxylin and eosin (H&E) as previously described [32]. Briefly, in each tissue section, thirty characteristic sites in the seminiferous tubules (STs) were randomly examined under a light microscope and graded by Johnsen’s score at ×400 magnification as described previously [32]. Spermatogenic cell density was analyzed by dividing the diameter of the seminiferous tubules by the thickness of the germinal cell layer.

To detect apoptotic cells in testicular seminiferous tubules, paraffin-embedded testicular sections were stained with a TUNEL assay kit (Dead End^TM^ Colorimetric TUNEL System for qualitative study; Promega, Madison, WI, USA) according to the manufacturer’s protocols. To assess apoptosis in cross sections of the testis, at least 30 seminiferous tubules were observed under a light microscope (×40 objective). The TUNEL-positive germ cells were calculated as the number of positive nuclei stained dark brown per seminiferous tubule, as described previously [24].

### 4.7. Immunohistochemistry Staining

Paraffin-embedded testis tissue sections were deparaffinized with xylene and rehydrated through a graded series of ethanol solutions. Then, the samples were heated in a 1× target retrieval solution, pH 6.0 (DAKO, Glostrup, Denmark). Endogenous peroxide activity was blocked with peroxidase-blocking solution (DAKO) for 15 min at room temperature (RT) and the sections were washed with 1× PBS buffer twice (5 min each). The tissue sections were then blocked with serum block solution for 10 min at room temperature (DAKO) and incubated with the primary antibodies StAR (1:100, D10H12, Cell Signaling Technology, Beverly, MA, USA), Gpx4 (1:100, Ab125066, Abcam, Cambridge, MA, USA), and cleaved caspase 3 (1:100, D175, Cell Signaling Technology, Beverly, MA, USA). The primary antibody was removed and the sections were washed again with 1× PBS twice (5 min each). Thereafter, a horseradish peroxidase (HRP)-labeled micropolymer conjugated with secondary antibody was applied (MP-7451, anti-rabbit IgG; vector Labs, Burlingame, CA, USA) for 1 h. Prior to incubation, the sections were rinsed with AEC(3-Amino-9-ethylcarbazole) substrate chromogens (SK-4205, ImmPACT AEC Peroxidase substrate; vector Labs, Burlingame, CA, USA). The slides were rinsed with deionized water for 3 min and counter-stained with hematoxylin. Finally, the slides were rinsed with distilled water prior to the addition of mounting medium (Abcam, Cambridge, MA, USA).

### 4.8. Malondialdehyde (MDA), Reactive Oxygen Species (ROS)/Reactive Nitrogen Species (RNS), and Antioxidant Enzymes Levels

Testis tissue malondialdehyde (MDA) levels were measured using an MDA assay kit as per the manufacturer’s instructions (NWLSSTM Malondialdehyde Assay kit; Northwest Life Science Specialties LLC, Vancouver, WA, USA). Briefly, MDA reacts with thiobarbituric acid forming a colored complex which was measured at an absorbance of 532 nm by a Spectra Max 180 (Molecular Devices, Sunnyvale, CA, USA). The MDA level is expressed as μmole MDA per mg of wet tissue. Testis tissue ROS/RNS levels were detected using an OxiSelect in Vitro ROS/RNS Assay Kit per the manufacturer’s instructions (STA-347, Cell Biolabs, Inc., San Diego, CA, USA). Briefly, absorbance was measured at 480 nm excitation/530 nm emission with a SpectraMax Gemini XS Fluorimeter (Molecular Devices, Sunnyvale, CA, USA) as described previously [20]. The activities of superoxide dismutase (SOD) (706002, Cayman Chemical, Ann Arbor, MI, USA), glutathione peroxidase (GPx) (703102, Cayman Chemical, Ann Arbor, MI, USA), and catalase (707002, Cayman Chemical, Ann Arbor, MI, USA) in whole testis tissue supernatants were determined using commercially available kits per the manufacturer’s instructions. SOD, GPx, and catalase activities are expressed per milligram of protein.

### 4.9. Western Blotting

Extraction of protein from testis tissue was conducted as described previously [32]. Briefly, the protein levels of apoptosis markers (pro-caspase-3, cleaved caspase 3, B-cell lymphoma 2 (Bcl-2), and BCL 2 associated X protein (Bax)), steroidogenic acute regulatory protein (StAR), glutathione peroxidase 4 (GPx 4), heme oxygenase-1 (HO-1), and nuclear factor erythroid 2-related factor 2 (Nrf2) in testis tissues were analyzed by Western blotting. Proteins (30–60 µg) were separated with 8 to 12% SDS-polyacrylamide gel electrophoresis. Thereafter, the gel was electro-transferred onto a PVDF membrane (#1620177, Bio-Rad, Hercules, CA, USA). The protein transfer membrane was blocked with 5% non-fat milk for 1 h at 40 °C and incubated with the following antibodies: pro-caspase-3, cleaved caspase 3, Bcl-2, Bax, StAR, and β-actin (Cell Signaling Technology, Beverly, MA, USA), GPx 4 (Ab125066, Abcam Cambridge, MA, USA), Nrf2 (sc-722, Santa Cruz Biotechnology, Dallas, TX, USA), and HO-1(ADI-SPA-896; Enzo Life Sciences, Farmingdale, NY, USA) in 5% non-fat milk overnight at 4 °C. Following incubation, the membranes were washed with TBS (Tris-buffered saline) containing 0.05% Tween 20 (TBST, pH 7.2) three times for 10 min each time and incubated with 1:5000 diluted anti-rabbit antibody (Cell Signaling Technology, Beverly, MA, USA) for 1 h at room temperature. The membrane was washed with TBST (10 min × 3 times). Specific binding was detected using an ECL kit (Amersham Bioscience, Piscataway, NJ, USA) under an ECL visualization system (Vilber Lourmat, France). Band intensities were analyzed using ImageJ software (NIH, Bethesda, MD, USA).

### 4.10. Statistical Analysis

All quantitative data are expressed as mean ± standard error of the mean (SEM). Statistical significance was determined using one-way analysis of variance (ANOVA) to compare the groups and Tukey’s test for post-hoc analysis (SPSS version 22; IBM, Armonk, NY, USA). A difference of *p* value < 0.05 was considered statistically significant. GraphPad PRISM 6.0 was used for graphical analysis (GraphPad Software, San Diego, CA, USA).

## 5. Conclusions

In conclusion, taken together, our data suggest that stress over a long period of time, perhaps surprisingly, is a cause of male infertility, and MOTILIPERM use should be considered as a novel strategy for the treatment of physiological and psychological stress-induced male infertility (Figure 5). MOTILIPERM treatment balances the redox state of testis tissue, upregulates testicular steroidogenesis, ameliorates spermatogenic impairment caused by immobilization-induced stress, and promotes antioxidant function in stressed rats to promote spermatogenesis. Furthermore, our findings suggest that MOTILIPERM induces the expression of anti-apoptotic proteins and downregulates the expression of pro-apoptotic proteins, and that modulation of the apoptotic process occurs through the Nrf2/HO-1 pathway. These findings suggest that MOTILIPERM represents a complementary medicine that could be used to counter stress-induced reproductive impairment.

## Figures and Tables

**Table 1:** Effect of MOTILIPERM on body weight and reproductive organ weight in immobilization stress-treated male Sprague Dawley (SD) rats.

Parameter	CTR	M 200	S	S + M 100	S + M 200
Body weight (sacrifice; g)	400.40 ± 9.15	395.90 ± 9.46	367.50 ± 5.99 *	362.33 ± 7.82	368.80 ± 3.1
Testis weight (g)	2.03 ± 0.03	2.01 ± 0.04	1.47 ± 0.15 *	1.46 ± 0.11	1.78 ± 0.07
Epididymis weight (g)	0.62 ± 0.02	0.62 ± 0.05	0.47 ± 0.02 *	0.48 ± 0.01	0.49 ± 0.01
Seminal vesicles weight (g)	1.66 ± 0.06	1.73 ± 0.04	1.49 ± 0.05	1.49 ± 0.06	1.52 ± 0.05
Prostate weight (g)	0.87 ± 0.04	0.83 ± 0.02	0.78 ± 0.02	0.72 ± 0.07	0.72 ± 0.03
Penis weight (g)	0.35 ± 0.01	0.34 ± 0.01	0.35 ± 0.07	0.32 ± 0.01	0.35 ± 0.01

Data are presented as mean ± SEM. The differences were tested by one-way ANOVA followed by Tukey’s post hoc test; *n* = 10 for each group. * *p* < 0.05 denotes significant difference compared to normal control (CTR) group. CTR: control; M 200: MOTILIPERM 200 mg/kg p.o.; S: stress by immobilization group; S + M 100: stress by immobilization + MOTILIPERM 100 mg/kg p.o.; S + M 200: stress by immobilization + MOTILIPERM 200 mg/kg p.o.; p.o.: per oral; ANOVA: analysis of variance; SEM: standard error of the mean.

## Supplementary Materials

Supplementary materials can be found at https://www.mdpi.com/1422-0067/21/13/4750/s1. Table S1. Radical scavenging activities of each component of MOTILIPERM on ABTS^+^ DPPH, deoxyribose degradation, and 2′-deoxyguanosine hydroxylation assays.

## Abbreviations

CTR	Control
M 200	MOTILIPERM 200 mg/kg
S	Immobilization stress
S + M 100	Immobilization stress + MOTILIPERM 100 mg/kg
S + M 200	Immobilization stress + MOTILIPERM 200 mg/kg
HPLC	High-performance liquid chromatography
SD	Sprague–Dawley
HPA	Hypothalamus–pituitary–adrenal
ER	Endoplasmic reticulum
Bcl-2	B-cell lymphoma 2
Bax	BCL 2 associated X protein
StAR	Steroidogenic acute regulatory protein
MDA	Malondialdehyde
SOD	Superoxide dismutase
ROS/RNS	Reactive oxygen species/reactive nitrogen species
GPx	Glutathione peroxidase
GPx 4	Glutathione peroxidase 4
Nrf2	Activating nuclear factor erythroid 2-related factor 2
HO-1	Heme oxygenase 1
GnRH	Gonadotropin-releasing hormone
LH	Luteinizing hormone
FSH	Follicle stimulating hormone
TUNEL	Terminal deoxynucleotidyl transferase-mediated (dUTP) nick-end labeling
H&E	Hematoxylin and eosin
ST	Seminiferous tubules
ANOVA	Analysis of variance
SEM	Standard error of the mean.
p.o	Per oral
