# Supplementary material for: MOTILIPERM Ameliorates Immobilization Stress-Induced Testicular Dysfunction via Inhibition of Oxidative Stress and Modulation of the Nrf2/HO-1 Pathway in SD Rats
Source: Int J Mol Sci. 2020 Jul 3;21(13):4750. doi: 10.3390/ijms21134750 (PMC7370033; doi:10.3390/ijms21134750)
Supplement: Supplementary file 1 [file ijms-21-04750-s001.pdf]

Supplementary Materials

Table S1. Radical Scavenging activities of each component of MOTILIPERM on ABTS+ DPPH, deoxyribose degradation, and 2′-deoxyguanosine hydroxylation assays.

Medicinal plants	Scavenging activity (SC50, ◎g/mL)			
	ABTS	DPPH	deoxyribose degradation[a]	2′-deoxyguanosine hydroxylation[b]
Morinda officinalis roots	> 500	> 200	16.73 ± 1.35	497.24
Allium cepa outer scales	4.51 ± 0.22	3.61 ± 0.39	30.48 ± 0.47	1.65
Cuscuta chinensis seeds	58.30 ± 0.23	32.06 ± 1.68	17.68 ± 0.99	> 1000

[a] mannitol was used as a positive control (SC_{50} value was 91.63 mM).
[b] mannitol was used as a positive control (SC_{50} value was 558.95 ◎M)

ABTS assay

The ABTS+ radical cation method [1] was modified to evaluate the free radical scavenging effect of each medicinal plant component of MOTILIPERM (*Morinda officinalis* roots, *Allium cepa* outer scales, and *Cuscuta chinensis* seeds). The ABTS reagent was prepared by mixing 5 mL of 7 mM ABTS with 88 μL of 140 mM potassium persulfate. After the mixture was kept in the dark at room temperature for 16 h to allow for the completion of radical generation, it was diluted with ethanol (1:44, v/v). To determine the scavenging activity, 190 ◎L of ABTS reagent was mixed with 10 ◎L of samples in a 96-well microplate and incubated at room temperature for 6 min. The final concentration of samples was prepared range from 0 (blank) to 100 ◎g/mL. After incubation, the absorbance was measured at 734 nm using a Bio-Rad Benchmark Plus microplate reader, and 75% ethanol was used as a control. The ABTS+ scavenging effect was calculated as: $(A_{control}–A_{sample}) / (A_{control}) X 100\%$. The SC_{50} values were obtained through extrapolation from the regression analysis. They denoted the sample concentration required to scavenge 50% of ABTS radicals. The antioxidant activities were evaluated using an SC_{50} value.

DPPH assay

The DPPH assay was performed as described [1]. The reaction mixture, containing an ethanolic solution of 200 μM DPPH (100 μL) and two-fold serial dilutions of the sample (dissolved in 100 μL ethanol, with sample amounts ranging from 0.001 to 10 mg/mL), was placed in a 96-well microplate and incubated at 37°C for 30 min. After incubation, the absorbance was read at 517 nm and the scavenging activity was determined by the following equation: % scavenging activity= $(A_{control} -A_{sample}) / (A_{control}) X 100$. The SC_{50} values were obtained using the same method as for the $ABTS^+$ radical scavenging assay.

Deoxyribose degradation assay

Deoxyribose degradation by hydroxyl radical (•OH) was carried out with slight modification

as described [2]. Samples were dissolved in absolute ethanol (0.001 – 10 mg/ml). An aliquot of sample solution was brought to 800 μl in phosphate buffer (25 mM, pH 7.2). Then, 50 μl of deoxyribose (3 mM), 100 μl of Na_2EDTA (1 mM), 100 μl of $FeCl_3$ (3.2 mM) and 100 μl of H_2O_2 (1 mM) were added. The reaction was initiated by mixing 100 μl of ascorbic acid (100 ◎M) and the total volume of the reaction mixture was adjusted to 800 μl with buffer. After incubation at 37 °C for 60 min, the reaction was terminated by 250 μl of trichloroacetic acid (2.8%, w/w). The color was then developed by addition of 125 μl of TBA (1%, in 50 mM NaOH aqueous solution) and heating in an oven at 100 °C for 15 min. The mixture was cooled and absorbance was measured at 532 nm (Bio-Rad Benchmark Plus microplate reader) against the buffer (as blank). The hydroxyl radical-scavenging activity was expressed as: $A_{control}$ -A_{sample}) / ($A_{control}$) X 100.

2′-Deoxyguanosine hydroxylation assay

The production of 8-hydroxy-2′-deoxyguanosine (8-OHdG) was performed as described [2]. The experimental conditions for the reaction of 2′-deoxyguanosine with •OH radicals were set as follows: E-tubes with a 1 l final volume, containing phosphate buffer pH 7.2 (25 mM), 2′-deoxyguanosine (150 μM), samples concentrations ranging from 0 (absence) to 10 mg/mL, $FeCl_3$ (100 μM), EDTA (100 μM), and ascorbic acid (200 μM)). To initiate the reaction, H_2O_2 (1 mM) was added and the samples incubated at 37°C for 60 min. Assays were also carried out with ethanol and mannitol as radical scavengers. Assays were performed in triplicate, and after incubation the samples were frozen until injection into HPLC the same day. The SC_{50} values were calculated by non-linear regression of the percentage of inhibition of 8-OHdG versus the concentration of samples

1. Kim, C.Y.; Lee, H.J.; Jung, S.H.; Lee, E.H.; Cha, K.H.; Kang, S.W.; Pan, C.-H.; Um, B.H. Rapid identification of radical scavenging phenolic compounds from black bamboo leaves using high-performance liquid chromatography coupled to an online ABTS+-based assay. *J. Korean Soc. Appl. Biol. Chem.* **2009**, *52*, 613-619.

2. Herraiz, T.; Galisteo, J. Hydroxyl radical reactions and the radical scavenging activity of β-carboline alkaloids. *Food Chem.* **2015**, *172*, 640-649.
